# Optimizing fracture resistance of endodontically treated maxillary premolars restored with preheated thermos-viscous composite post-thermocycling, a comparative study. Part I

**DOI:** 10.1186/s12903-024-03959-7

**Published:** 2024-03-02

**Authors:** Heba B. Abdel-Maksoud, Bassem M. Eid, Mai Hamdy, Heba M. Abdelaal

**Affiliations:** 1 Restorative Dentistry Department, Faculty of Dentistry, Suez Canal University, Ismailia, 41611 Egypt; 2 Restorative Dentistry Department, King Abdulaziz University, P.O. Box 80209, Jeddah, 21589 Saudi Arabia; 3 Restorative Dental Sciences Department, College of Dentistry, Gulf Medical University, Ajman, UAE; 4 Endodontic Department, Faculty of Dentistry, King Salman International University, El Tur, Egypt; 5 Endodontic Department, Faculty of Dentistry, Suez Canal University, Ismailia, 41611 Egypt; 6 Restorative Dentistry Department, Faculty of Dentistry, Ain Shams University, Cairo, Egypt

**Keywords:** Viscalor Bulk, Pre-heated thermos viscous, Endodontically treated teeth, Fracture resistance, Bulk fill, fiber-reinforced

## Abstract

**Purpose:**

This research aimed to investigate fracture resistance of endodontically treated maxillary premolars restored using preheated thermo-viscous and fiber-reinforced bulk fill resin composite, in vitro.

**Methodology:**

Sixty sound human maxillary premolars were selected and divided randomly into 6 groups of ten teeth each (*n* = 10). Group 1; is the positive control with sound unprepared teeth (P), Group 2; is the negative control in which Mesio-occluso-distal (MOD) cavities were left unrestored (N), Group 3; includes the teeth restored by incremental packing with conventional nanohybrid composite (ChP), Group 4; includes teeth restored with short fiber reinforced bulk fill composite (EF), Group 5; includes teeth restored with preheated thermo-viscous bulk fill composite (VB), and Group 6; includes teeth restored using packable bulk fill composite (XF) Tested restorative materials were bonded with a universal adhesive in self-etch mode. Teeth were kept in distilled water for 24 h at 37 °C proceeded by thermocycling (5- 55 °C, 1200×). Teeth were then exposed to compressive load till fracture at a crosshead speed of 1 mm/min. One-way ANOVA followed by Tukey post-hoc test was implemented to compare between more than two groups in non-related samples. The significance level was established at α = 0.05 for both tests.

**Results:**

Intact teeth significantly recorded the highest fracture resistance values among all groups. A significant difference was recorded among all the tested groups, with the EF recording the highest values, followed by the VB group then the XF group and ChP that recorded the lowest data. Negative control premolars significantly recorded the lowest fracture.

**Conclusions:**

After thermocycling, endodontically treated maxillary premolars restored with pre-heated thermos-viscous composite did not exhibit an increase in fracture resistance. Notably, our findings indicate that short fiber-reinforced composite demonstrated significantly higher fracture resistance compared to other types of composites assessed in this study. This suggests the potential superiority of short fiber-reinforced composite in enhancing the overall structural integrity of endodontically treated teeth subjected to occlusal forces.

## Introduction

Fracture resistance of endodontically treated teeth (ETT) is considered a very important factor that decides the success of treatment. They are inherently mutilated and weak due to the minimal remaining tooth structure, in other words, they lost a major part of their structure due to excessive decay or fracture [1, 2]. The loss of tooth tissues results from extensive caries removal and/or after old restoration replacement [3] before endodontic treatment, together with different procedural steps during the treatment [4]. This, in turn, affects the biomechanical behavior of the teeth. Studies have shown that the decreased stiffness is due to cavity preparations and removal of decay rather than the physical changes and dehydration of dentin resulted from the endodontic treatment [5, 6]. Moreover, being non-vital limits the sensory response during peak loads and predominantly renders them much more susceptible to fracture, [7] which is non-restorable in the majority of cases [8] and even catastrophic. Unfortunately, tooth fracture is considered to be the third most common cause of tooth loss following dental caries and periodontal diseases [9].

Since the fracture resistance of restored teeth is greatly influenced by the type of tooth [10], in addition to the fact that maxillary premolars are known to have the highest incidence [11] and greatest susceptibility [12] to fracture under occlusal loading in the oral cavity, therefore, testing the fracture resistance of the tooth-restoration complex will be informative in studying the restorative material behavior. This is due to their occlusal anatomy [13, 14] which is characterized by accentuated cusp inclinations. In addition to the narrow cervical thickness they have, and the concavity present on the mesial aspect of the root, a radicular groove present on the palatal aspect of the buccal root prejudices them to more cusp fractures, wedging action, as well as splitting [15–17].

Again, by knowing that the fracture resistance of restored teeth is mostly influenced by the cavity size and expansion [10, 18], evaluation of the fracture resistance is beneficial on mesio-occluso-distal (MOD) prepared cavities. Clinical experience has shown that maxillary premolars with deep MOD cavities are susceptible to fracture when eccentric forces are applied [19]. They are clinically relevant by simulating a common scenario in dental practice. They involve removing a specific amount of tooth structure which results in stress concentration in the remaining part. Evaluation of fracture resistance aids in understanding the behavior of different restorative materials or techniques under the exceptional oral environmental conditions. The loss of tooth walls, particularly the marginal ridges, causes a significant decrease in tooth fracture resistance more than access cavity preparations [18, 20] and an aggressive decrease in toughness eightfold more than access cavity [6].

Proper selection of the final restorative material greatly affects the fracture resistance of root canal treated teeth [21–23]. The resistance to fracture of the material itself is very crucial to ensure reliable performance of the tooth-restoration complex [24]. In the multifactorial oral environment, the used restorative material should help the remaining tooth material in non-vital teeth to stand against massive forces to which they are subjected, otherwise catastrophic fractures might result. Several restorative materials were used a long time ago to directly restore the coronal part of these teeth. Resin composites were the first choice for decades, because of aesthetics, availability, easy application, and of course for serving in conservation of tooth structure [25]. They are classified according to their filler size, the delivery method, as well as the presence or absence of fibers. Every class of resin composites has advantages and like any other dental material has some drawbacks. Conventional and bulk fill resin composites are two major competitors in the restorative field. The latter was introduced to achieve all advantages of the conventional type with less complex techniques. It ensures perfect contacts, very good mechanical properties, enhanced stress response, and faster speed of introducing the material into the prepared cavity [26]. These criteria in turn decreases the number of clinical steps and the effort exerted by the dental practitioner [27]. Since the root canal treatment is counted a relatively long dental procedure, therefore, a restorative material that helps to decrease the time of the dental visit required to finaly restore the ETT is considered to be very beneficial to dental practitioner. A newly introduced preheated thermos-viscous bulk fill composite that aids in time saving is used in this research. The manufacturer asserted that it combines the characteristics of flowable composite during application and sculpt ability of packable composites. This facilitates single-phase restorations with a minimum of working steps and thus reducing the dental treatment time. All of the above-mentioned factors render the selection of a suitable material to restore root canal treated teeth critical.

Despite a lot of research discussing the restoration of these teeth, an ideal material is not yet clear to the dental clinicians. Therefore, the objective of this research is to evaluate the effect of restoring maxillary premolars with the newly introduced pre-heated thermo-viscous resin composite that uses near-infrared technology in comparison to alternative bulk fill resin composite materials in vitro.

## Materials and methods

### Materials

Four commercial composites were investigated. The material’s types, lot numbers, abbreviations, composition, filler Load (wt%), and manufacturer recommendations are presented in Table 1.

**Table 1:** The materials’, types, lot numbers, abbreviations, composition, filler Load (wt%) and manufacturer recommendations

**Type**		**Name (LOT, Abbreviation)**	**Mode of Insertion**	**Composition**	**Filler Load / wt %**	**Manufacturer-** **Recommended** **Curing**	
Preheated thermo-viscous		VisCalor bulk (2,039,302, VB)	Pre-heat	Matrix: amine, BHT, bis-GMA, CQ, dimethacrylate Filler: SiO2 nanofillers (20–40 nm), barium-aluminum-silicate glass particles (1.2 μm)	83	Voco; Cuxhaven, Germany-	
Bulk Fill Hybrid		X-tra fil (2,218,545, XF)	Conventional	Inorganic fillers in a methacrylate matrix, Bis-GMA, UDMA, TEG-DMA	83.		
SFRC Flowable		EverX Flow Bulk (2,112,021, EF)	Injectable	Matrix; Bis-MEPP, TEG-DMA and UDMA Fillers; mix of short E-glass fibres and particle fillers, mostly barium glass.	70	GC Europe	
Nanohybrid		CharmFil Plus (1,122,157, ChP)	Conventional	Martrix; Bis-GMA, TEGDMA, UDMA Barium glass 0.5 μm	> 70	Dentkist Inc, Korea	
Flowable universal nano-hybrid		Grandio Flow (2,202,596, GF)	Conventional	Bis-GMA, TEG-DMA, HEDMA	80.2	Voco; Cuxhaven, Germany-	
Dual curing Universal adhesive		Futurabond M+ (2,122,733, FB)	-----	HEMA (10-25%), BIS-GMA (10-25%), ethanol (10-25%), acidic adhesive monomer (2.5-5%), UDMA (2.5-5%), catalyst (≤ 2,5%), pyrogenic silicic acids (≤ 2,5%)	---		

BHT: butylhydroxytoluene, bis-GMA: bisphenol-A-glycidyl-dimethacrylate;

CQ: camphorquinone; HEMA: 2-hydroxylethyl methacrylate, TEG-DMA: triethylene glycol dimethacrylate; UDMA: urethane dimethacrylate

### Sample preparation

The research was given approval from the Research Ethics Committee at Faculty of dentistry King Abdul Aziz University (138-12-22), according to the guidlines of Declaration of Helsinki of World Medical Association. Sample size calculation used 0.05 alpha value and 80% power for detection of a difference of 25% (PiFace, http://homepage.stat.uiowa.edu/~rlenth/Power/ (seen on 24 June 2022). A common standard deviation in a single group was calculated to be 18%. The approximate size of the sample in each of the groups should be at least 9 [28]. Intact sixty human maxillary premolars were carefully collected according to ethical guidelines. All teeth were carefully examined using a light microscope to ensure they were free from any cracks or fractures. They were thoroughly washed and cleaned from any calculus or tissue remnants. Teeth were initially accurately measured and organized by size. They were then examined for similarity using a digital micrometer with the following dimensions: buccolingual width: 8.47–10.59 mm; mesiodistal width: 6.38–8.19 mm. Teeth were kept in distilled water at 37 °C before use, for up to a month. Afterward, they were randomly allocated to the assigned groups.

### Assignment of prepared samples

Random teeth were grouped into six groups *n* = 10; Positive control (Group 1) in which teeth were left unprepared (P), Negative control (Group 2) in which Mesio-occluso-distal (MOD) cavities were prepared and left with no restoration (N), the Conventional (Group 3) with teeth restored with conventional composite CharmFil Plus (ChP); bulk fill (Group 4) where teeth were restored using short fiber reinforced bulk fill composite, everX Flow Bulk (EF); bulk fill (Group 5) in which teeth were restored using preheated thermo-viscous bulk fill composite, Viscalor Bulk (VB); and bulk fill (Group 6) with teeth restored using packable bulk fill composite XtraFil (XF).

### Cavity preparation

Diamond bur (Diatech, Germany) was used to prepare MOD cavities on the Mesio-occluso-distal surface with the following dimensions: the depth of the occlusal cavity was 4 mm, the width at the isthmus was 1/3 of intercuspal distance, the proximal box was 2/3 the bucco-palatal width and the gingival floor 1.0 mm above cemento-enamel junction [29]. After every five cavity preparations, the bur was changed. A periodontal probe and a caliper were regularly used throughout the preparation procedures to measure the cavity depth and dimensions. Then, each and every tooth was put individually in a container with a specific mark. Only one operator was responsible for performing all the cavity preparations to ensure standardization of the cavity dimensions.

### Restorative procedures

#### Endodontic preparation and restoration

An Endodontic consultant prepared all premolars for standardization issues. A high-speed handpiece was used to prepare endodontic access cavities. A #2 diamond round bur by Dentsply, Tulsa, OK, USA was used for pulp chamber roof penetration, and then a tapered cylinder bur was used for extension. All overhangs were then removed. Files, size 10 K (Mani Inc, Japan) were introduced into root canals. By subtracting 0.5 mm from the length, the working length was recorded. ProTaper rotary instruments (Dentsply-Maillefer, Ballaigues, Switzerland) till master apical rotary size F2 (#25) were used to prepare the canals, using 2 mL of 5.25% sodium hypochlorite for irrigation in-between files.

5 mL of 17% ethylenediamine tetraacetic acid (Pulpdent Corporation, USA) was used for rinsing prepared root canals, then the final rinse was completed with 5 mL of distilled water. Drying was completed using paper points. Then, ProTaper F2 gutta-percha and AH Plus (Dentsply DeTrey, Konstanz, Germany) epoxy resin-based sealer were utilized by single-cone technique for filling. The excess was eliminated from the coronal region. The access cavities were sealed with a temporary filling (Coltosol, Coltene, Brazil) and all tested samples were kept in 100% humidity for one week for the sealer to set. A Tofflemire metal matrix band/retainer was used to encircle each tooth. To ensure proper fit and alignment of the band with the cavity edges, external support was provided by the use of a low-fusing compound [30].

#### Cavity restoration

Teeth in restored groups were bonded with Futurabond M + in self-etch mode. The adhesive was shaken to mix the components together. It was applied uniformly over the internal walls of the cavity using a micro brush. A 3-way syringe was used at a 20 cm distance for dryness. Then, it was cured for 10 s from the occlusal, mesial, and distal directions for a total of 30 s (1200 mW/cm2, LED Curing Light, Ivoclar Vivadent, Liechtenstein). The tip was placed 2 mm distance away from the cavity surface.

Regarding all bulk fill groups, cavities were restored according to the assigned type of composite according to the manufacturer’s instructions. For the Bulk fill group-VB; cavities were restored using a preheated thermo-viscous composite VB. For every cavity, fresh compule was preheated and applied simultaneously by the novel all-in-one device VisCalor Dispenser. It uses near-infrared technology (NIR) to heat composite in 30 s providing a fixed temperature for a particular time interval (150 s) [31]. The temperature inside each preheated compule reached 65 °C and was monitored with a digital thermometer (TES-1300, Taiwan). The material was then directly placed into the cavity with no need to remove it from the warmer.

For the Bulk fill group-XF; cavities were restored with XF bulk-fill composite. A 4 mm increment of the composite was extruded from the tube and used to bulk fill the cavity using a plastic instrument.

For the Bulk fill group-EF; cavities were restored with a 4 mm layer of EF using bulk injection.

Then, for the Conventional group-ChP, two increments of 2 mm each, were used to fill the cavity by incremental packing technique using plastic instruments.

For all restored groups, any excess material was removed, and a glass slide was put on the occlusal surface to pack the material uniformly. This step is important for the structural integrity of the composite while curing. Restorations were photoactivated occlusally for 10s and, after the removal of the Tofflemire matrix, it was light cured mesially and distally for 10 s each; total curing time: 30s. During the restoration of the cavities, a Radiometer was used to regularly check light intensity during sample preparation.

Finally, the finishing took place with a diamond burr. For standardization issues, all procedural steps were completed by only one specialist. The specimens were stored in distilled water at 37 °C for 24 h to allow for entire polymerization.

### Thermocycling

Restored teeth were put for thermocycling between 50 and 55 °C (dwell time 30 s) for 1200 x (Thermocycler, Mechatronic, Germany). ISO TR 11,405 recommends a minimum of 500 thermal cycles for short-term aging of dental materials [32]. In addition, Gale and Darvell postulated that a total of 10,000 cycles may potentially correspond to an estimated duration of one year in terms of in vivo functionality, whereas a range of 20 to 50 cycles seemed comparable to a single day [33]. Accordingly, 1200 cycles were equivalent to a maximum of 60 days of aging.

### Mechanical testing

Teeth roots were coated with 0.2–0.3 mm wax. Every tooth was embedded in a horizontal direction in an acrylic resin block till 1 mm apical to CEJ, with its long axis perpendicular to the bottom of the block. Boiling water was used to melt the wax and replaced it with polyvinyl siloxane impression material to simulate periodontium. Compression was performed in a universal testing machine (Instron 3345, Lloyd, UK). A 6 mm diameter steel sphere was used to contact the buccal and palatal cusps slopes to put an occlusal load in a perpendicular direction at a crosshead speed of 1 mm/min. This continued till fracture occurred and was noted in newtons (N) using computer software BlueHill Instron [34]. 

### Statistical analysis

Statistical analysis was performed with IBM-SPSS Statistics Version 20 for Windows. Data were assessed for normality by Kolmogorov-Smirnov and Shapiro-Wilk tests, data revealed parametric (normal) distribution. One-way ANOVA followed by Tukey post-hoc test was used to compare between more than two groups in non-related samples. The significance level was set at α = 0.05 for both tests.

**Table 2:** Means and Standard Deviations (SDs) of Fracture Resistance of Groups (*n* = 10)

Groups	Mean	SD
P	1345.21	36.31
N	104.41	8.35
ChP	405.41	29.25
XF	658.90	73.65
VB	927.82	71.68
EF	1193.53	89.45

*p*-value < 0.001*

*Significant (*p* < 0.05), ns non-significant (*p* > 0.05)

## Results

**Fig. 1:**
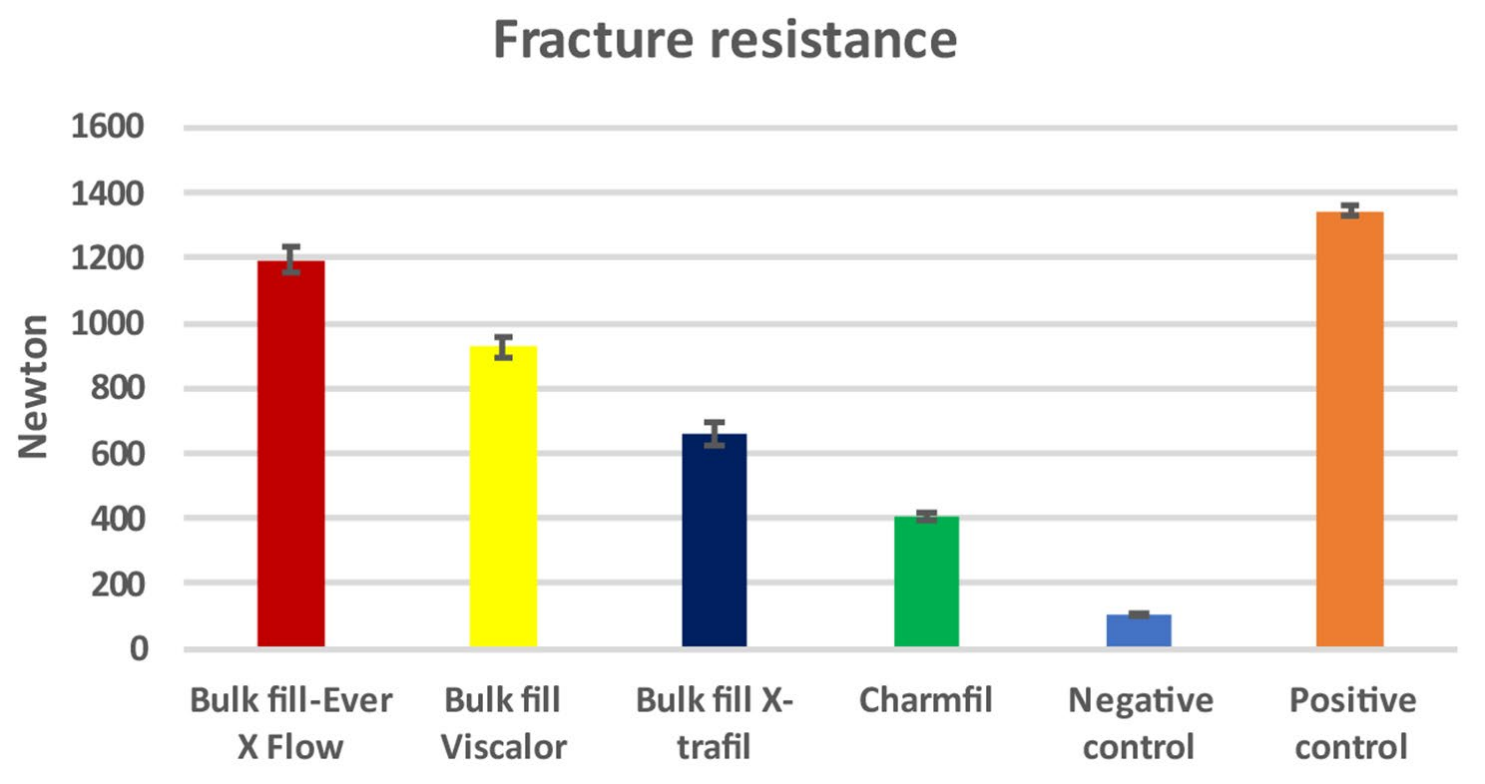
Mean fracture resistance of each material expressed in *N*

Mean and standard deviation values of fracture resistance for every group are accessible in Table 2/Fig. 1. Positive control P, (1345.21 ± 36.31 N) reported the significantly highest fracture resistance (*p* < 0.05) among all tested groups, followed by SFRC EF, (1193.53 ± 89.54 N), then preheated thermo-viscous composite VB (927.82 ± 71.68 N), followed by bulk fill hybrid XF (658.90 N), and finally the conventional ChP, (405.41 ± 29.25 N). The negative control N showed the significantly lowest values (104.41 ± 8.35 N) (*p* < 0.05).

## Discussion

During the restoration of endodontically treated teeth, the selection of the dental material used for the final restoration of the coronal part is considered a critical decision. Two main issues are to be addressed, the inherent weakness of these teeth and the ideal material for which dental clinicians are searching. The final restoration should provide satisfactory protection, and restore the teeth’ biological, aesthetic, and mechanical integrity [35]. Besides, the teeth’ reduced elasticity comprises a major issue and should be taken into consideration. Since this ideal material is non-existent, manufacturers are trying to develop a restorative material that meets the required criteria. Bulk fill, as well as fiber-reinforced composites, were introduced to restore ETT as direct restorations are the widely used restoration of these teeth. Resin composites are characterized by excellent strength; however, they don’t have toughness. Modifications of bulk fill composites continued to improve to enhance the material’s adaptation to cavity walls with the preservation of superior mechanical properties. From these modifications is the preheated thermos-viscous VB. This research aimed to use the currently introduced thermo-viscous resin composites to restore EET to increase their resistance to fracture after thermocycling. The maxillary premolars were used as representatives for the posterior region where reinforcing the remaining tooth structure after an endodontic procedure can be challenging.

Regarding cusp stiffness, it was reported that one marginal ridge might cause a mean loss of 46% if it is lost [36]. Besides, maxillary premolars have sharp cusp inclines making them more prone to fracture [13]. MOD cavities represented ETT with significant tooth loss and lowered resistance to fracture [37, 38].

When the first bulk-fill composite was released, it required the placement of an extra layer of conventional composite. However, other materials in the same class that were introduced later were claimed by manufacturers to be placed with no need for that layer. Others had to be capped with conventional RBC for aesthetics and physical characteristics [39]. The change in the technique of restoration with materials of the same category slightly confuses dental practitioners who thought that all materials of the same class should behave the same. To the best of our knowledge, and up to date there are many studies investigating resistance to fracture of bulk fill restored endodontically treated teeth, bulk fill flowable, and fiber-reinforced composites comparing them with conventional ones [23, 40–43]. However, limited, or no published studies compared the fracture resistance of root canal-treated teeth using preheated thermos-viscous composites using infra-red technology among other types of composites. The null hypothesis tested was that there would be no difference in fracture resistance of endodontically treated premolars restored with different types of bulk fill composites.

The results of the present study reported significant differences in the fracture resistance between all tested groups. Therefore, the null hypothesis was rejected. The ChP group, in which EET were restored with an incremental technique resulted in the lowest values when compared to the rest of the tested groups. This finding was expected in agreement with many studies that reported that although the direct restorations packed in an incremental technique were introduced to adhesive dentistry to reduce the internal stresses, they are not the perfect option to reinforce EET against fracture, especially in the case of MOD cavities [44, 45].

The addition of different fibers’ orientations mixed with resin composite materials significantly increases the fracture resistance of ETT teeth. This is precisely what gave rise to our study where, in the EF group, which is SFRC, the highest fracture resistance records were reported among all the groups tested. This comes in accordance with a research that stated increased fracture resistance of endodontically treated premolars when flowable bulk-fill composites were used in the final restoration [46]. Manufacturers assert that the substance itself prevents crack growth. This was confirmed in some studies which stated that the short millimeter scale of composite with haphazardly placed E-glass fibers with a unique intermingling polymer produced a crack stopper [44, 47–49]. When comparing the materials physical properties to bulk fill and conventional composites, Garoushi et al.in 2013 found that EF performed better than the rest of evaluated materials in terms of fracture resistance [50]. In addition to this result, they showed the lowest shrinkage strain. They explained the resulted data by stating that polymer matrix plasticization by linear polymer chains of PMMA in cross-linked matrix of bisphenol A diglycidyl ether dimethacryalate– triethylene glycol diemthacrylate, represented the primary cause. It raised fracture toughness and stress transmission between polymer matrix and fibers, enhancing the strengthening effect of the fibers incorporated., More recently in 2023, a research reported a significant increase in the SFRC restoring ETT [44] and explained that by stating that the toughening ability of the material which contains short millimeter-scale randomly orientated E-glass fibers with a unique semi-interpenetrating polymer network structure that act as a crack stopper.

Contrary to our results, there is a non-significant difference in fracture toughness between SFRC and classical composites used for Class II MOD cavities in molars. Only when oblique layering technique was utilized, increase in fracture resistance was observed [41, 51]. The difference might be attributed to the difference between the tested materials as well as the critical difference in sample preparation. The latter is very important as in the current study prepared teeth were used, with a percentage of compliance, however, in some studies materials specimens were prepared in fabricated molds. The contradiction might be also due to the difference in the adhesive system used. The bonding used has a crucial role in bond achievement at the cavity-restoration interface. This will consequently have an impact on the final restoration’s fracture resistance. Moreover, fiber addition had non-significant effect on increasing the fracture resistance of composites in another study by Seidy et al., in 2023. They stated that there was a clear trend towards greater fracture resistance and restorable fractures only when using this material with the diagonal layering technique [40]. 

Regarding VB group, teeth restored with the preheated thermos viscous composite recorded significantly lower fracture resistance values than EF. It was claimed by manufacturers to combine characteristics of flowable composite during application and sculpt ability of packable composites. This facilitates single-phase restorations with a minimum of working steps. It has 1.44% volume shrinkage and 4.6 MPa shrinkage stress which is considered superior within bulk-fill composite materials with enhanced mechanical stability [31]. Elevating composites temperature when restoring cavities enhances adaptation to cavity walls as a result of decreased viscosity of composite [52]. Considering the findings of the present study, Scepanovic et al. found VB restorations showed potential to improved resistance against challenges facing dentinal adhesion, even after thermomechanical loading. They stated that this preheated composite failed to achieve good marginal adaptation in class V restorations although the material was used as manufactures instructions [53].

The significant decrease in fracture resistance of VB might be explained by the insufficient monomer-polymer conversion of the material after curing because of immobilisation, steric isolation, and gelation [54]. This would eventually an outcome that leaves behind unreacted monomers in the polymer chain, depressing the composite’s biocompatibility, producing cytotoxic reactions, and reducing the mechanical characteristics, such as wear, flexural strength, and tensile qualities. In summary, pre-heating of VB would not help in increasing monomer-polymer conversion and didn’t increase the dentin composite bond strength. This might clarifies how significantly lower values results in the present study. In contrast, VB mechanical properties were found better when compared to those applied without preheating [55].

A significant difference was recorded between VB and EF. Although VB has higher filler content 83% by weight more than EF 70%, it showed a significantly lower fracture resistance. This outcome may be explained by that the short fibers used in the fillers of EF composite help in increasing the materials fracture resistance. The results were in accordance with Nayar et al. [56] who reported that E-glass fibers could sustain strength characteristics within extremely difficult conditions and that they were insensitive to some extent to moisture contamination as well as chemicals. These composites were used in large cavities as a base in areas of stress concentration. The E-glass fiber is composed of aluminoborosilicate glass with lesser amount of alkali oxides.

In XF group, ETT showed a significantly lowered fracture resistance values when compared to the tested groups. XF flowable bulk fill composite was evaluated in several studies and reported acceptable results in terms of degree of conversion and microhardness with no differences existed between bottom and top of the restoration as well as highest modulus of elasticity reported [57]. Regarding the filler content, it was expected that XF with higher percent of filler content (83% by weight) would report significantly higher fracture resistance than EF (70% by weight), as the compressive strength is dependent on the percent of filler content. However, the fiber composition of EF might be the reason behind the increase in fracture resistance that is like the higher filler content XF. Variables like form, size, and filler particles distribution also affects the mechanical strength, elastic modulus and hardness of resin composites. Besides, the material’s flexure strength, water uptake, and biocompatibility were reported to be like conventional RBCs in other studies.

Another study reported non-significant difference between bulk fill and bulk fill flowable composites [57]. Although polymerization shrinkage PS of both composites is different. PS might not be the only reason behind the contraction stresses [58], the similarity between both composites might be due to lower flexural modulus and delayed contraction rate [59].This went in agreement with Toz et al. who reported no difference between bulk fill flowable and bulk fill composites in fracture resistance of ETT [60]. Besides, Yasa et al. found no difference between nanohybrid composite, bulk fill flowable composite, and SFRC in the absence of retention slots [61].

As the use of bulk fill restoratives is demanding nowadays, especially those with easier application they are clinically recommended in terms of fracture resistance to restore endodontically treated teeth. However, the results of the present invitro study should be validated with additional clinical studies to take into consideration the functional and parafunctional forces. In endodontically treated teeth, and after been claimed by manufacturers that they have excellent adaptation, further studies should take place to test the adaptation of these bulk fill materials to the floor of the pulp chamber even without the need to add any intermediate material between them and the root canal filling material.

The best that we can tell, limited in-vitro research studied the different mechanical properties of preheated thermo-viscous composites used to restore endodontically treated teeth. Therefore, clinical investigations are required to support the use of these composites and much more importantly relate them to the clinical scenario.

## Conclusion

Within the confines of the present study, all restored teeth reported lower fracture resistance values when compared to the intact teeth group. After thermocycling, endodontically treated maxillary premolars restored with pre-heated thermos-viscous composite did not exhibit an increase in fracture resistance. Notably, our findings indicate that short fiber-reinforced composite demonstrated significantly higher fracture resistance compared to other types of composites assessed in this study. This suggests the potential superiority of short fiber-reinforced composite in enhancing the overall structural integrity of endodontically treated teeth subjected to occlusal forces.

## Data Availability

Raw data is available upon request from the corresponding author.
